# ROCK as a Therapeutic Target of Diabetic Retinopathy

**DOI:** 10.1155/2010/175163

**Published:** 2010-06-21

**Authors:** Ryoichi Arita, Yasuaki Hata, Tatsuro Ishibashi

**Affiliations:** Department of Ophthalmology, Graduate School of Medical Sciences, Kyushu University, 3-1-1 Maidashi, Higashi-Ku, Fukuoka 812-8582, Japan

## Abstract

The increasing global prevalence of diabetes is a critical problem for public health. In particular, diabetic retinopathy, a prevalent ocular complication of diabetes mellitus, causes severe vision loss in working population. A better understanding of the pathogenesis and the development of new pharmacologic treatments are needed. This paper describes the relevance between Rho/ROCK pathway and the pathogenesis of diabetic retinopathy from its early to late stages. Moreover, the therapeutic potential of ROCK inhibitor in the total management of diabetic retinopathy is discussed.

## 1. Introduction

The growing prevalence of diabetic retinopathy (DR), the common ocular complication of diabetes mellitus, is a critical problem for global public health [1, 2]. Early nonproliferative stages of DR are characterized by blot hemorrhages and vascular abnormality such as retinal vascular microaneurysms or hyperpermeability which could cause diabetic macular edema (DME). Proliferative diabetic retinopathy (PDR), later stage of the diseased state, causes neovascularization, vitreous hemorrhages, preretinal fibro-vascular proliferation, and tractional retinal detachment. While visual acuity is not always affected in early stages, progression of the disease leads to severe vision loss.

Panretinal photocoagulation (PRP) and vitreoretinal surgery remain the primary therapeutic strategies for progressed DR. However, PRP is destructive to the retina and accompanied by adverse effects such as decreased visual acuity, increased risk of macular edema, and pain [3, 4]. Moreover, recent advances in vitreous surgery cannot always achieve a satisfying visual acuity [5]. Now it is at a point where new alternative and adjunctive agents from the earlier stages are urgently required because of overwhelming patient's physical and economic burdens of these treatments.

The various clinical findings in earlier DR stages are related to endothelial damage secondary to increased leukocyte adhesion mediated through adhesion molecules, intercellular adhesion molecule-1 (ICAM-1), and leukocyte *β*_2_-integrins (CD18/CD11a and CD18/CD11b) [6–8]. In addition, the critical mechanism of this leukocyte-induced endothelial damage is the interaction of endothelial Fas with Fas ligand, expressed on adherent leukocytes in diabetic rats [9] and DR patients [10].

Recent accumulating evidences indicate that vascular endothelial growth factor (VEGF) plays a critical role in pathogenesis of both DME and neovascularization in PDR [11, 12]. Clinical studies with anti-VEGF antibodies are potentially useful strategy and improve outcome for treating DR [13]. However, the adaptation is only for progressed states and there is a possibility of systemic adverse complications such as hypertension, cerebrovascular accidents, and myocardial infarcts by anti-VEGF therapy [14]. 

Meanwhile, neovascularization and proliferative vitreoretinopathy (PVR) are hallmark of the later DR stages. VEGF-induced endothelial migration and proliferation is essential process for angiogenesis. ERK1/2 signaling promotes the proliferative activities of endothelial cells in angiogenic processes [15, 16]. Moreover, endothelial migration is mediated by Rho-kinase (ROCK) pathway which activates remodeling of endothelial F-actin cytoskeleton [17]. 

Our recent findings indicated that hyalocytes, a known resident macrophage in the cortical vitreous under physiological conditions, appeared to be involved in the pathogenesis of PVR associated with a cicatricial contraction of proliferative membranes in PDR [18–20]. The expression of *α*-smooth muscle actin (*α*-SMA) and phosphorylation of myosin light chain (MLC) in hyalocytes, which is associated with stress-fiber formation and contractile rings, are facilitating cell contraction [21, 22]. We demonstrated that vitreous from patients with PDR significantly promoted expression of *α*-SMA and phosphorylation of MLC, and enhanced contraction of hyalocyte-containing collagen gels, compared with vitreous from patients with nondiabetic controls [23].

 In this paper, we first place the Rho/ROCK pathway plays a critical role in diabetic retinal microvasculopathy, neovascularization, and tractional retinal detachment associated with a cicatricial contraction of proliferative membranes. We then describe our current knowledge about ROCK inhibition as a new strategy in the total management of DR from its early to late stages.

## 2. Involvement of Rho/ROCK Pathway in the Pathogenesis of Diabetic Retinopathy

**Figure 1:**
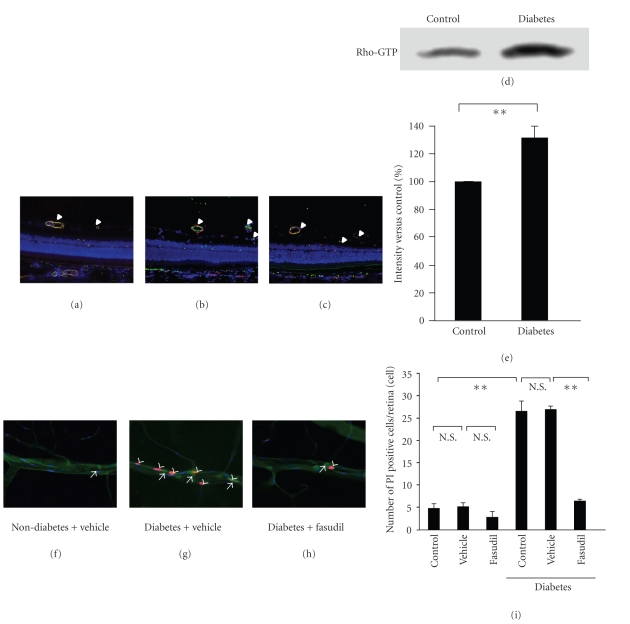
Rho/ROCK activation in retinal vessels. In immunohistochemical analysis, RhoA (a), ROCK1 (b) and ROCK2 (c) were detected in retinal vessels. Yellow (white arrowhead) indicates double-stained vasculature (magnification: ×400). (d and e) The levels of Rho-GTP were significantly higher in streptozotocin induced-diabetic rat bretinas, compared with those in nondiabetic control, detected by Rho pull-down assay. Average signal intensities are quantified and expressed as percentage of the ratio of control (**P < .01, *n* = 5 each). Prevention of leukocyte-induced retinal endothelial damage by fasudil. (f–i) *In vivo* visualization of adhering leukocytes (green, concanavalin A lectin) and injured endothelial cells (red, propidium iodide (PI)) and endothelial nuclei (blue, DAPI) in rat retinas. PI positive cells (white arrowhead) widely coincided with adherent leukocytes (white arrow). The number of PI positive cells per retina was significantly higher in the diabetic animals, compared with the nondiabetic controls. Fasudil caused a significant reduction in the number of PI positive cells in the retinas of the diabetic animals, compared with the vehicle-treated controls (**P < .01, N.S., not significant, *n* = 5 each).

Recent studies have revealed that small GTP-binding protein Rho and its target protein ROCK are implicated in the important physiological roles such as cell adhesion and migration mediated through MLC phosphorylation [19, 24]. Rho activity is also increased in bovine aortic endothelial cells treated with high glucose [25], and involved in the pathogenesis of renal and aortic complications during diabetic states [26, 27]. Furthermore, we revealed that Rho/ROCK pathway is activated in retinal microvessels during diabetes (Figures 1(a)–1(e)). 

Rho/ROCK pathway promotes leukocyte adhesion to the microvasculature by affecting the expression and function of adhesion molecules, including ICAM-1 [28, 29] and integrins [30]. Moreover, ROCK causes firm adhesion through the activation of ezrin, radixin, and moesin in endothelial cells, which jointly form the anchoring structures for leukocytes' integrins [31, 32]. These findings suggest that elevated activity of the Rho/ROCK pathway is involved in the pathogenesis of diabetic microvasculopathy mediated through leukocyte adhesion.

Rho/ROCK signaling is also involved in the pathogenesis of VEGF-induced angiogenesis. In endothelial cells ROCK pathway plays a critical role in VEGF-induced endothelial migration by regulating stress fiber formation associated with MLC phosphorylation [17, 33]. Moreover, recent *in vivo* studies have also demonstrated that the ROCK pathway plays a critical role in angiogenesis [34, 35].

Previously we showed TGF-*β*2 contributes to transdifferentiation of hyalocytes into *α*-SMA positive myofibroblast-like cells that causes hyalocyte-containing collagen gel contraction [19]. Moreover, we revealed that TGF-*β*2, overexpressed in the vitreous and contractile membranes of PDR patients, activates ROCK pathway and forms stress fibers and contractions mediated through ROCK activation [20, 23]. These results suggest the central role of ROCK in the cicatricial contraction of proliferative membrane in PDR patients.

## 3. Therapeutic Strategy in Diabetic Retinopathy by ROCK Inhibition

### 3.1. ROCK Inhibition Ameliorates Diabetes-Induced Microvascular Damage

Fasudil, a potent and selective ROCK inhibitor, is relatively safe and effective in the treatment of cardiovascular disease including cerebral and coronary vasospasm, angina, hypertension, and heart failure with no serious adverse side effect in fasudil-treated patients [36]. In our animal experiments, intravitreal injection of fasudil did not cause apparent electrophysiological or morphological changes in retinal tissues at least within its effective concentrations [23]. However, several adverse effects such as hepatic function abnormal, intracranial hemorrhage, and hypotension have been reported [37, 38]. We thus need further examination regarding the safety and adverse effects of ROCK inhibitor before its clinical use in the field of intraocular diseases.

 Nevertheless, we recently could reveal the therapeutic potential of fasudil in the management of earlier stages of DR. Treatment with intravitreal injection of fasudil significantly decreased retinal leukocyte adhesion in diabetic rat mediated through reduction of ROCK activation. Moreover, fasudil effectively suppressed endothelial damage, even when leukocytes firmly adhered to the endothelium (Figures 1(f)–1(i)). This suggests that fasudil directly causes endothelial protection in addition to its impact on leukocyte adhesion. Rho/ROCK inactivates endothelial nitric oxide synthase (eNOS) in human umbilical venous cells [39]. eNOS generates physiological levels of nitric oxide (NO), a potent vasodilator [40] and antiapoptotic factor [41, 42]. Fasudil treatment almost completely reversed the decreased eNOS activity in diabetic rat retinas. In addition, the protective effect of fasudil on microvascular endothelial cells was significantly blocked by NOS inhibition with L-NAME, without apparent effect on leukocyte adhesion *in vitro*. These findings suggest that fasudil has a direct endothelial protective potential through induction of physiological levels of NO, synthesized by eNOS.

### 3.2. Antiangiogenic Properties of ROCK Inhibitor

We demonstrated that ROCK inhibitor could inhibit VEGF-elicited bovine retinal capillary endothelial cell (BREC) migration and proliferation *in vitro* and corneal neovascularization *in vivo*. A ROCK inhibitor fasudil had inhibitory effect on BREC migration with a scratch-wound assay. Moreover, fasudil could inhibit VEGF-induced BRECs [^3^H]-thymidine incorporation and ERK1/2 phosphorylation, whose activity indicates the proliferative activities of endothelial cells in angiogenic processes [15]. *In vivo*, fasudil strongly attenuated VEGF-induced corneal neovascularization in a corneal pocket assay [43].

### 3.3. ROCK Inhibition Suppresses Critical Contraction of Proliferative Membrane

**Figure 2:**
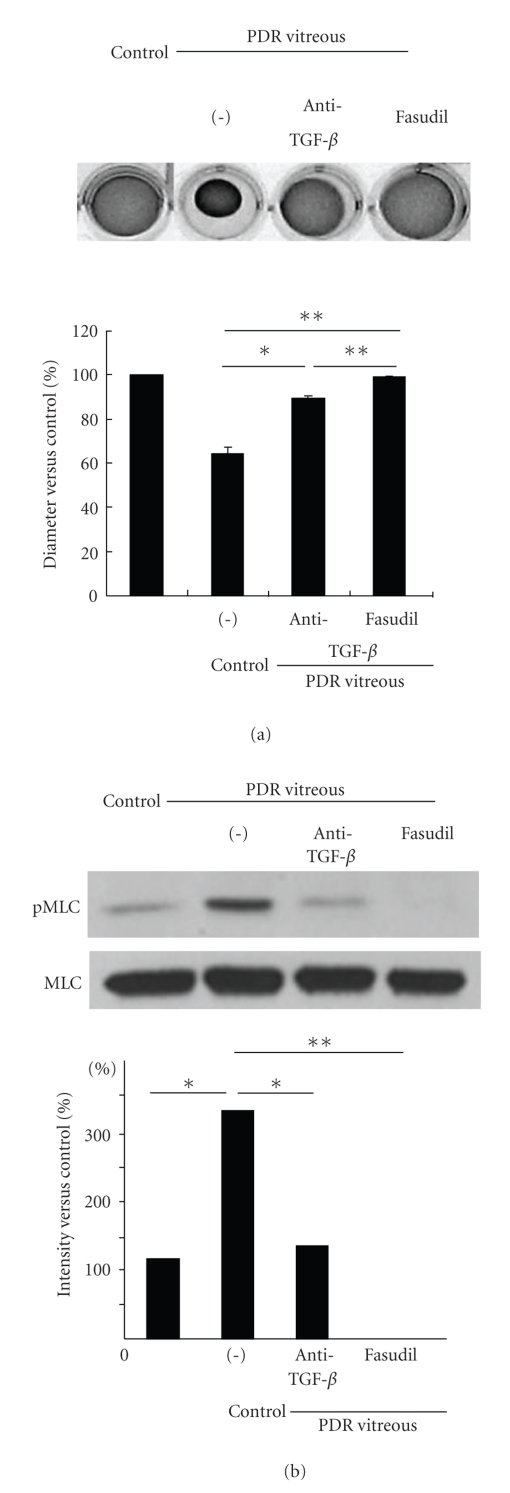

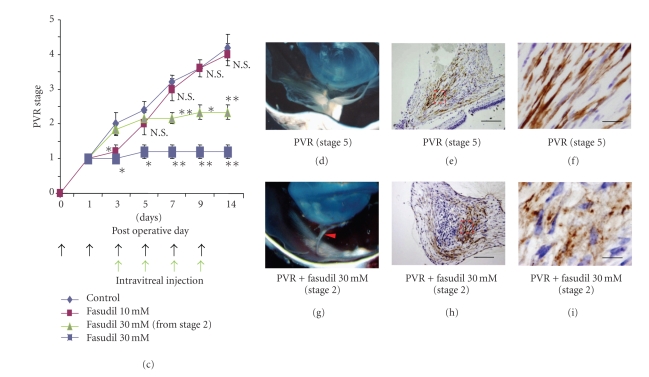
Impact of fasudil on PDR vitreous-induced collagen gel contraction and MLC phosphorylation. After pretreatment with or without anti-TGF-*β* mAb or fasudil, hyalocytes were stimulated with vitreous with PDR. (a) In hyalocyte-containing collagen gels, fasudil almost completely suppressed the contraction of collagen gels treated with PDR vitreous. The diameter of the gels was measured and statistically analyzed (*P < .05; **P < .01; NS, not significant, *n* = 3 each). (b) Western blot analysis was performed to detect phosphorylated MLC (pMLC). Fasudil abolished MLC phosphorylation, induced by PDR vitreous. Lane-loading differences were normalized by MLC. Signal intensities were quantified and expressed as percentages of the pMLC/MLC ratio compared with control (*P < .05; **P < .01, *n* = 3 each). Experimental PVR in rabbit eyes. (c) Therapeutic potential of fasudil in reducing the progression of experimental PVR. PVR was classified into six stages (0–5). Rhombus, vehicle (*n* = 5); purple square, fasudil 10 *μ*M (*n* = 5); trigone, fasudil 30 *μ*M from stage 2 (*n* = 6); blue square, fasudil 30 *μ*M (*n* = 5) (*P < .05, **P < .01, not significant versus vehicle). (d) Tractional retinal detachment because of formation and cicatricial contraction of preretinal proliferative membrane was observed by stereomicroscopy in vehicle-treated eyes (stage 5 PVR). (g) In contrast, intravitreal membranes adhered to the retina without causing retinal detachment (arrowhead) in 30 *μ*M fasudil-treated eyes with stage 2 PVR. Micrographs depict *α*-SMA expression (brown) in preretinal proliferative membrane with stage 5 PVR (e) and stage 2 PVR (h) by immunohistochemical analysis. (Scale bar, 200 *μ*m). (f and i) Magnified images of (e) and (h),respectively, (Scale bar, 10 *μ*m).

We could also demonstrate the therapeutic potential of ROCK inhibitor fasudil in the management of later stages of DR. In hyalocyte-containing collagen gels assay, fasudil almost completely abolished the PDR vitreous-induced collagen gel contraction mediated through the suppression of MLC phosphorylation (Figures 2(a) and 2(b)). In experimental PVR rabbit model, fasudil also effectively disrupted *α*-SMA organization and blocked contraction of proliferative membrane (Figures 2(c)–2(i)). 

Statins, inhibitors of the 3-hydroxy-3-methyl-glutaryl- (HMG-) CoA reductase, are widely used to reduce endogenous cholesterol synthesis and improve hypercholesterolemia [44]. By inhibiting HMG-CoA reductase, statins also block ROCK activation mediated through the mevalonate pathway [45].

We demonstrated that simvastatin almost completely inhibited vitreous-induced contraction of the collagen gels in *exvivo* and proliferative membrane in experimental PVR model mediated through ROCK inhibition [46]. Our results indicate that ROCK inhibition suppresses PVR progression in later DR stages.

### 3.4. Other Beneficial Effects of Fasudil on Retinal Tissue during Diabetes

#### 3.4.1. Vasodilatory Property and Improvement of Hemodynamics in the Retinal Vessels

**Figure 3:**
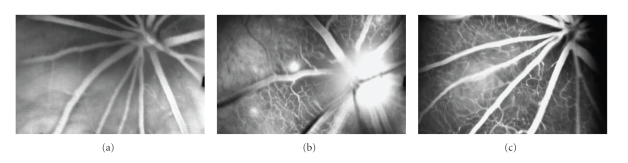
Contractile impacts of ROCK activation in rat retinal vasculature. Intravitreal injections ofllysophosphatidic acid (LPA), Rho activator, were performed into rat's eyes over a period of 1 minute with a 33-gauge needle. The final intraocular concentration of LPA was 20 *μ*M. We monitored the retinal fluorescein with a scanning laser ophthalmoscope ((a) no injection, (b) 5 minutes after injection, (c) 10 minutes after injection). Intravitreal injection of induced sever retinal vessel contraction.

Development of chronic retinal ischemic state aggravates diabetic retinopathy. Rho/ROCK activation plays an important role in the pathogenesis of vasoconstriction, such as cerebral and coronary spasm [47] or hypertension [48], by NO-dependent mechanisms. We could show that intravitreal injection of lysophosphatidic acid, a potential Rho activator, induced severe retinal vessel constriction (Figures 3(a)–3(c)). Recent studies suggested that ROCK inhibitor fasudil improved hemodynamic states in human [49], and also dilated rat retinal vessels, and increases blood flow [48]. These results suggest that fasudil has preventable benefit on retinal ischemia during diabetes through improvement of hemodynamics in the retinal vessels.

#### 3.4.2. Retinal Neuroprotective Effect of Fasudil

Retinal ischemia secondary to DR causes functional and irreversible damage not only in retina vasculature but also in retinal neuronal cells. Chronic loss of neuronal cells from the inner retina by increasing the frequency of apoptosis reduces the thickness of the nerve fiber layer in diabetic retina [50]. Impaired retinal electrophysiology and neurodegeneration have been shown in diabetic patients [51, 52]. Recent studies revealed that Rho/ROCK pathway also seems to be associated with the pathogenesis of this neuronal damage. Abnormal activation of the Rho/ROCK pathway is important in the pathogenesis of several neurological diseases [53]. In rat retina, Rho/ROCK pathway is also involved in N-methyl-D-aspartat-induced neurotoxicity in the rat retina. [54]. These studies suggest that ROCK inhibitor would protect against neuronal damage by acting directly on neurons. In fact, the ROCK inhibitor Y-27632 increases regeneration of retinal ganglion cell in the rat optic nerve crush model [55]. Moreover, ROCK inhibition attenuates ischemia-induced retinal neuronal cell death by inhibiting leukocytes extravasation and release of proinflammatory cytokines such as TNF-*α* or IL-6 *in vitro/vivo* [56, 57]. These data suggest that inhibition of Rho/ROCK pathway leads to neuroprotective effect and promote retinal cell survival during diabetes.

## 4. Conclusion

**Figure 4:**
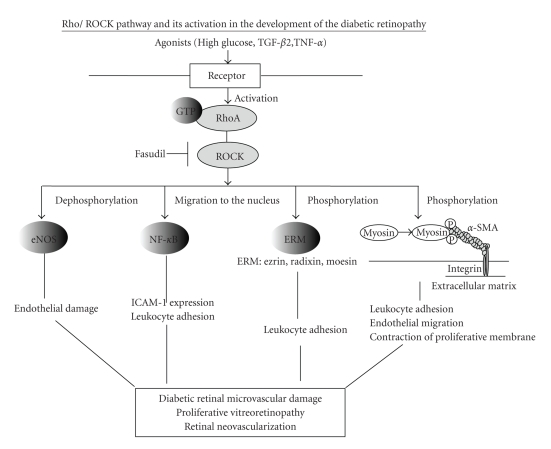
Rho/ROCK pathway and its activation in the development of diabetic retinopathy. RhoA is a small GTP-binding protein, and Rho-kinase (ROCK) is its target protein. Glucose, TNF-*α*, and TGF-*β*, elevated in diabetic serum or vitreous, activate Rho/ROCK pathway in endothelial cells or hyalocytes. ROCK activation induces endothelial damage mediated through inactivation of endothelial nitric oxide synthase (eNOS), which has endothelial protective potential. Moreover, ROCK causes firm leukocyte adhesion through the increase of ICAM-1 expression and activation of ezrin, radixin, and moesin (ERM) in endothelial cells. ROCK also has important roles such as leukocyte adhesion, endothelial migration, and contraction of proliferative membrane mediated through myosin light chain (MLC) phosphorylation in diabetic retinopathy. These findings suggest that elevated activity of the Rho/ROCK pathway is involved in the pathogenesis of diabetic microvascular damage, proliferative vitreoretinopathy, and retinal neovascularization.

Rho/ROCK pathway is involved throughout the pathogenesis of DR, particularly in diabetic retinal microvasculopathy, neovascularization, and tractional retinal detachment associated with cicatricial contraction of preretinal proliferative membranes (Figure 4). Since we must consider frequent intravitreal injections as administration method due to a short biological half-life time of the compound in the vitreous cavity, we are considering intravitreal implantation of a slowly releasing drug-delivery system. For preventable benefit on progressing retinal microvascular damage and keeping good visual acuity, timing of intravitreal fasudil implantation prefers when early clinical DR findings such as microaneurysm begin to appear. In addition, pre- and postoperative intravitreal implantations for active PDR patients with proliferative membrane are also considered to be effective for prevention of PVR and tractional retinal detachment. Whereas further basic and clinical studies to reveal the effectiveness and safety of ROCK inhibitor are needed for clinical use in the field of eye diseases, ROCK inhibition might become a novel therapeutic strategy in the total management of DR from its early to late stages.
